# Genetic factors underlying tacrolimus intolerance after liver transplantation

**DOI:** 10.3389/fimmu.2022.944442

**Published:** 2022-09-30

**Authors:** Yuan Liu, Rui Wang, Peizhen Wen, Wenbin An, Jinxin Zheng, Tao Zhang, Pengshan Zhang, Haoyu Wang, Fan Zou, Hui Pan, Junwei Fan, Zhihai Peng

**Affiliations:** ^1^ Department of General Surgery, Shanghai General Hospital, Shanghai Jiao Tong University School of Medicine, Shanghai, China; ^2^ Department of General Surgery, Xiang’an Hospital of Xiamen University, School of Medicine, Xiamen University, Xiamen, China; ^3^ Organ Transplantation Institute of Xiamen University, Fujian Provincial Key Laboratory of Organ and Tissue Regeneration, School of Medicine, Xiamen University, Xiamen, China; ^4^ Department of Nephrology, Ruijin Hospital, Institute of Nephrology, Shanghai Jiao Tong University School of Medicine, Shanghai, China; ^5^ Department of Organ Transplantation, The Second Affiliated Hospital of Guangzhou Medical University, Guangzhou, China

**Keywords:** *CYP3A5*, liver transplantation, tacrolimus intolerance, tacrolimus, insulin resistence

## Abstract

**Background:**

Tacrolimus (FK506) is the cornerstone of immunosuppression after liver transplantation (LT), however, clinically, switching from FK506 to cyclosporine (SFTC) is common in LT patients with tacrolimus intolerance. The aim of this study was to investigate the genetic risk of patients with tacrolimus intolerance.

**Methods:**

A total of 114 LT patients were enrolled in this retrospective study. SNPs were genotyped using Infinium Human Exome-12 v1.2 BeadChip, and genome-wide gene expression levels were profiled using Agilent G4112F array.

**Results:**

SFTC was a potential risk factor of dyslipidemia (OR=4.774[1.122-20.311], *p* = 0.034) and insulin resistance (IR) (OR=6.25[1.451-26.916], *p* = 0.014), but did not affect the survival of LT patients. Differential expression analysis showed donor *CYP3A5*, *CYP2C9*, *CFTR*, and *GSTP1*, four important pharmacogenetic genes were significantly up-regulated in the tacrolimus intolerance group. Twelve SNPs of these four genes were screened to investigate the effects on tacrolimus intolerance. Regression analysis showed donor rs4646450 (OR=3.23 [1.22-8.60] per each A allele, *p* = 0.01), donor rs6977165 (OR=6.44 [1.09-37.87] per each C allele, *p* = 0.02), and donor rs776746 (OR=3.31 [1.25-8.81] per each A allele, *p* = 0.01) were independent risk factors of tacrolimus intolerance.

**Conclusions:**

These results suggested that SFTC was a potential risk factor for dyslipidemia and IR after LT. Besides, rs4646450, rs6977165, and rs776746 of *CYP3A5* might be the underlying genetic risks of tacrolimus intolerance. This might help transplant surgeons make earlier clinical decisions about the use of immunosuppression.

## Graphical Abstract

A total of 114 LT patients were enrolled in this retrospective study. SNPs were genotyped using Infinium Human Exome-12 v1.2 BeadChip, and genome-wide gene expression levels were profiled using Agilent G4112F array. Switching from FK506 to cyclosporine (SFTC) was a potential risk factor of dyslipidemia and insulin resistance. Regression analysis showed donor rs4646450, donor rs6977165, and donor rs776746 were independent risk factors of tacrolimus intolerance.

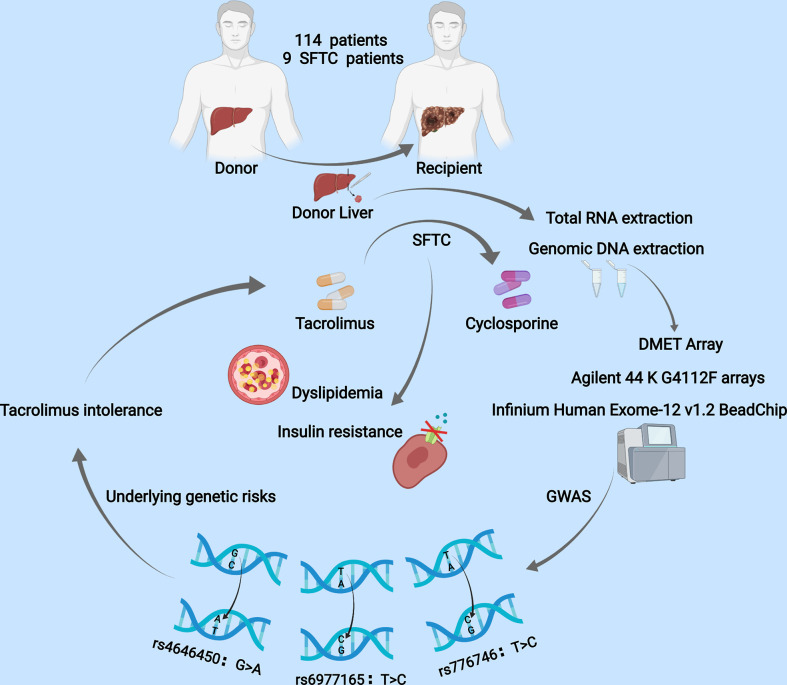

## Introduction

With the success of liver transplantation (LT) operation and the use of immunosuppression, the long-term survival of recipients may be limited by metabolic syndrome (1, 2). Metabolic syndrome after transplant mainly consists of diabetes, dyslipidemia, hypertension, and obesity, the core role is insulin resistance (1, 3). To some extent, we believe that more and more metabolic syndrome incidence is because of immunosuppression, especially tacrolimus (FK506) using, which has a narrow therapeutic index and wide interindividual pharmacokinetic variability, which easily causes acute rejection and toxicity (4–6).

Interestingly, we note that some recipients of rapid tacrolimus metabolism must be switched to another immunosuppressive regiments for both economic and safety reasons. Hence, we wonder about the genetic factors behind switching from tacrolimus to cyclosporine (SFTC), as well as the influence of SFTC on prognosis. In this study, we integrate the multi-omics data of our transplant recipient with exome chip and transcriptome data to identify the reason for patients’ intolerance to tacrolimus and achieve individualized medicine.

## Materials and methods

### Patients

A total of 114 patients who underwent orthotopic liver transplantation between Jan 2015 and Dec 2017 at the First People’s Hospital, affiliated to Shanghai Jiao Tong University School of Medicine, were enrolled in our study. All of them were Han Chinese and received tacrolimus-based immunosuppressive regimens. Nine patients in our study cohort experienced SFTC and One hundred five patients did not experience SFTC (NSFTC). Patients with fasting glucose of more than 110 mg/dl or 6.11 mmol/L were deemed as candidates for insulin resistance according to the definition of metabolic syndrome. (7)

### Ethics statement

Liver transplants are derived from donations after cardiac death (DCD). Does not contain the donated liver obtained from the executed prisoners. The study was approved by the Ethics Committee of Shanghai First Hospital affiliated with Shanghai Jiao Tong University. These methods are based on the Helsinki Declaration and its subsequent amendments or similar ethical standards.

### Data hoarding

The pharmacological parameters of tacrolimus include daily dose and drug blood trough concentration. Blood samples for tacrolimus monitoring were collected before morning administration. Blood trough concentration of tacrolimus was detected by the Pro-TracTMII tacrolimus ELISA kit (Diasorin, Stillwater, MN, USA) with a microparticle enzyme immunoassay (ELx 800NB analyzer, BioTek, Winooski, VT, USA). The results of which were then recorded and the tacrolimus trough blood concentration/weight-adjusted-dose ratios (CDR, (ng/ml)/(mg/kg)) in the different periods were calculated.

### Gene expression data

Gene expression microarray data used for differential expression analysis has been described in our previous study (8). The expression data of the four samples were evaluated for array intensity distributions (box plots). The expression intensity values were log2 transformed. Further analysis was performed using the R package ‘limma’. The very important pharmacogenetic (VIP) genes (*n* = 63) were obtained from the Pharmacogenomics Knowledgebase (http://www.PharmGKB.org).

### Genomic DNA isolation and Genotyping

The genomic DNA of the donor was extracted from liver tissues (stored at -80°C) using AllPrep DNA/RNA Mini Kit (Qiagen, Hilden, Germany). The genotypes of all samples were determined by Infinium Human Exome-12 v1.2 BeadChip (EXON chip) containing 333,445 exon variations. The *CYP3A5* genotype was selected from the Drug Metabolizing Enzymes and Transporters (DMET) chip (9). The recipients analyzed with propensity score matching (PSM) did not have dyslipidemia and insulin resistance before LT, and then some recipients developed dyslipidemia and insulin resistance, and some did not after LT.

### Statistical analysis

The SFTC and NSFTC samples used in the expression analysis were selected based on the results of the PSM, eliminating interference due to age, gender, and primary disease. Tacrolimus CDRs were normalized by logarithmic transformation (logCDR). Patients’ survival was calculated with Kaplan-Meier survival curves and compared using the log-rank test. The effects of immunosuppression regimen conversion on postoperative complications were evaluated by logistic regression analysis. Genotype data analysis and quality control were performed by using PLINK software (10). Groups were compared using the Wilcoxon-Mann-Whitney test. All statistical analyses were performed using statistical software R (version 3.5.2). All statistical tests were the two-sided test, and *p* < 0.05 was considered statistically significant.

## Results

### Effects of SFTC on survival and prognosis of patients after LT

**Figure 1:**
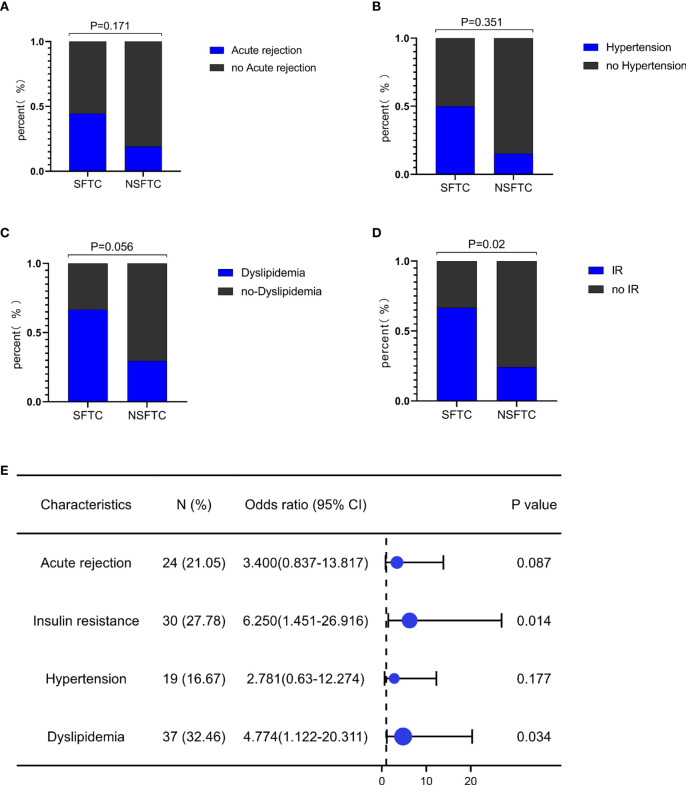
Switching from FK506 to cyclosporine (SFTC) associated with the poor prognosis. **(A)** SFTC increased the incidence of insulin resistance; **(B)** SFTC might increased the incidence of dyslipidemia; **(C)** SFTC might increased the incidence of hypertension; **(D)** SFTC might increased the incidence of acute rejection; **(E)** The forest plot showed the correlation of SFTC and complications. P less than 0.05 means statistical significance.

There were 95 males and 19 females with a mean age of 47.4 ± 9.0 years in our study, the other detailed clinical information is in **Table 1**. Among these, nine patients experienced SFTC. As **Figures 1A-D** shows, SFTC was associated with the incidence of insulin resistance (IR) (*p* = 0.02), as well as might correlated with the incidence of dyslipidemia (*p* = 0.056), acute rejection (*p* = 0.171), and hypertension (*p* = 0.351) after LT. Besides, the forest plot showed that SFTC increased the incidence of IR (*p* = 0.014, OR=6.25[1.451-26.916]), and dyslipidemia (*p* = 0.034, OR=4.774[1.122-20.311]) (**Figure 1E**). However, there is no statistically significant difference in patient survival between the SFTC group and NSFTC group (*p* = 0.31, **Figure S1**). The associations between post-transplant complications and survival are shown in **Figure S2**.

**Table 1:** Characteristics of patients enrolled in this study.

Recipient	*N* = 114
Age (years) (mean ± SD)	47.4 ± 9.0
Gender, male/female (n)	95/19
Child-Pugh score (mean ± SD)	7.1 ± 2.1
MELD score (mean ± SD)	11.8 ± 5.6
Tacrolimus logCDR, median (IQR)	
Week 1	5.5 (5.0-6.1)
Week 2	4.9 (5.0-5.3)
Week 3	4.7 (4.3-5.2)
Week 4	4.8 (4.4-5.3)
Post-transplant complications (n)	
Acute rejection	24
New-onset hypertension	19
New-onset dyslipidemia	37
Insulin resistance	30

Categorical variables are shown as n and continuous variables that are displayed as mean±standard deviation or median (IQR). IQR, interquartile range. logCDR, logarithmically transformed trough blood concentration/weight-adjusted-dose ratios. The threshold of Insulin resistance in the definition of metabolic syndrome.

### Identification of genes influencing the SFTC process

**Figure 2:**
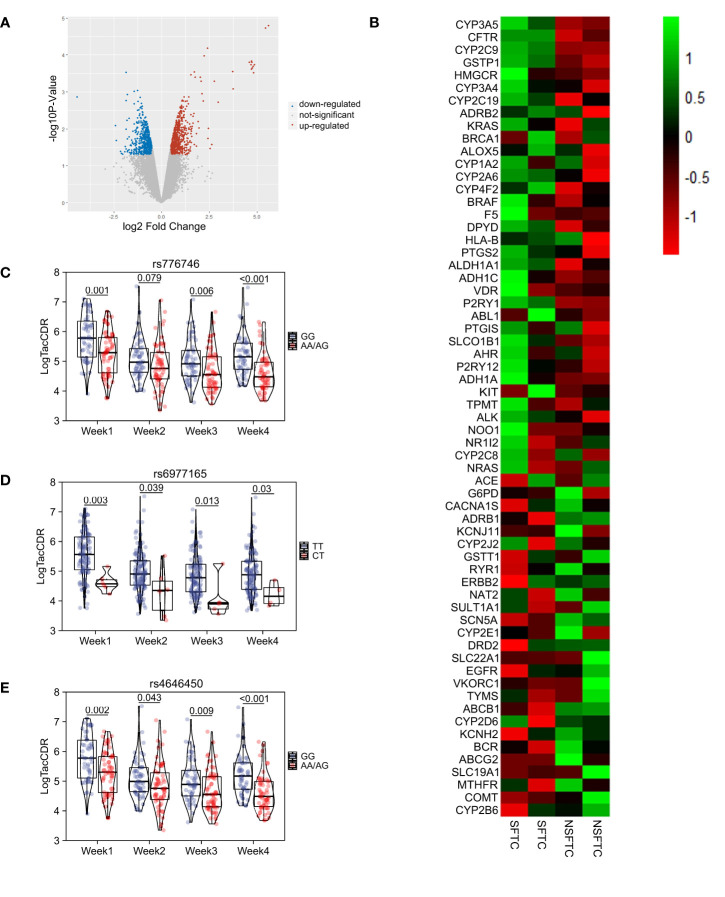
The screening of genes influencing the process of Switching from FK506 to cyclosporine (SFTC). **(A)** Volcano plot showed the difference gene between SFTC and non-SFTC patients. Red dots represent upregulated genes, and blue dots represent downregulated genes; **(B)** Heatmap showed the very important pharmacogenetic genes expression level between SFTC and non-SFTC patients; **(C)** Comparison of tacrolimus pharmacokinetics at first four weeks in different genotype groups (AA/AG vs GG) of donors’ *CYP3A5* rs4646450; **(D)** Comparison of tacrolimus pharmacokinetics at first four weeks in different genotype groups (CT vs TT) of donors’ *CYP3A5* rs6977165; **(E)** Comparison of tacrolimus pharmacokinetics at first four weeks in different genotype groups (AA/AG vs GG) of donors’ *CYP3A5* rs776746. P less than 0.05 means statistical significance.

Gene expression analysis was conducted using donor liver transcriptome data between the SFTC group and NSFTC group after PSM. The results identified 675 and 596 transcripts expression levels significantly up-regulated and down-regulated, respectively (**Figure 2A**). To identify genes involved in tacrolimus intolerance, we only focused on those significant genes involved in the metabolism of or response to one or several drugs, which have been summarized as the very important pharmacogenetic (VIP) genes in the Pharmacogenomics Knowledgebase. The expression level of the VIP genes was shown in **Figure 2B**. We found four VIP genes (*CYP3A5*, *CFTR*, *CYP2C9*, *GSTP1*) were significantly up-regulated in the SFTC group. Thus, our study was focused on these four differentially expressed VIP genes (VIPDEGs).

### Twelve SNPs of four VIPDEGs were included in this study

The SNPs of VIPDEGs were screened from the EXON chip and the *CYP3A5* rs776746 genotype was selected from the DMET chip. MAF and HWE of each SNP in donors were calculated, respectively. The genetic variants with a minor allele frequency (MAF) of more than 0.05 were included in the study because of the small sample size. At last, twelve SNPs of VIPDEGs were included in this study (**Table 2**). Allele frequencies were all in Hardy–Weinberg equilibrium (*p >*0.05).

**Table 2:** SNPs of VIPDEGs included in the study.

SNP	Gene	CHR	A1	A2	MAF	HWE (P)
rs28365085	*CYP3A5*	7	G	A	1.75%	1.00
rs55965422	*CYP3A5*	7	G	A	0.88%	1.00
rs4646450	*CYP3A5*	7	A	G	30.09%	0.38
rs6977165	*CYP3A5*	7	C	T	2.63%	1.00
rs776746	*CYP3A5*	7	A	G	29.65%	0.50
rs1800073	*CFTR*	7	T	C	0.88%	1.00
rs141723617	*CFTR*	7	C	T	1.32%	1.00
rs121909046	*CFTR*	7	G	A	1.75%	1.00
rs213950	*CFTR*	7	A	G	39.47%	0.05
rs75789129	*CFTR*	7	G	A	5.26%	1.00
rs1057910	*CYP2C9*	10	C	A	3.51%	1.00
rs1695	*GSTP1*	11	G	A	20.18%	0.39

SNP, single-nucleotide polymorphisms; CHR: chromosome number; A1, minor allele code; A2, major allele code; MAF, minor allele frequency; HWE, Hardy–Weinberg Equilibrium; CYP3A5 means cytochrome P450 3A5; CFTR means CF Transmembrane Conductance Regulator; CYP2C9 means Cytochrome P450 family 2 subfamily C member 9; GSTP1 means Glutathione S-Transferase Pi 1; P less than 0.05 means statistical significance.

### The association between the donor VIPDEGs gene polymorphisms and SFTC

The correlation between SFTC and donor SNP was calculated using PLINK (**Table 3**). Among the twelve SNPs, three SNPs of *CYP3A5* were shown statistical significance. They were rs4646450 (OR=3.23 [1.22-8.60] per each A allele, *p* = 0.01), rs6977165 (OR=6.44 [1.09-37.87] per each C allele, *p* = 0.02), rs776746 (OR=3.31 [1.25-8.81] per each A allele, *p* = 0.01), respectively. However, the other SNPs were not found to be related to SFTC (*p* > 0.05).

**Table 3:** Comparison of genotype distribution between SFTC and NSFTC groups.

SNP	Gene	Genotype frequency, n				P	OR (95% CI)
rs28365085	*CYP3A5*		GG	GA	AA	0.20	4.06(0.40-41.16)
		SFTC	0	1	8		
		NSFTC	0	3	102		
rs55965422	*CYP3A5*		GG	GA	AA	0.68	0
		SFTC	0	0	9		
		NSFTC	0	2	103		
rs4646450	*CYP3A5*		AA	AG	GG	0.01	3.23(1.22-8.60)
		SFTC	2	6	1		
		NSFTC	6	46	52		
rs6977165	*CYP3A5*		CC	CT	TT	0.02	6.44(1.09-37.87)
		SFTC	0	2	7		
		NSFTC	0	4	101		
rs776746	*CYP3A5*		AA	AG	GG	0.01	3.31(1.25-8.81)
		SFTC	2	6	1		
		NSFTC	6	45	53		
rs1800073	*CFTR*		TT	TC	CC	0.68	0
		SFTC	0	0	9		
		NSFTC	0	2	103		
rs141723617	*CFTR*		CC	CT	TT	0.61	0
		SFTC	0	0	9		
		NSFTC	0	3	102		
rs121909046	*CFTR*		GG	GA	AA	0.55	0
		SFTC	0	0	9		
		NSFTC	0	4	101		
rs213950	*CFTR*		AA	AG	GG	0.34	1.59(0.61-4.18)
		SFTC	3	3	3		
		NSFTC	20	41	44		
rs75789129	*CFTR*		GG	GA	AA	0.30	0
		SFTC	0	0	9		
		NSFTC	0	12	93		
rs1057910	*CYP2C9*		CC	CA	AA	0.62	1.71(0.20-14.69)
		SFTC	0	1	8		
		NSFTC	0	7	98		
rs1695	*GSTP1*		GG	GA	AA	0.82	1.14(0.36-3.65)
		SFTC	0	4	5		
		NSFTC	6	30	69		

OR, estimated odds ratio (for A1); CI, confidence interval; CYP3A5 means cytochrome P450 3A5; CFTR means CF Transmembrane Conductance Regulator; CYP2C9 means Cytochrome P450 family 2 subfamily C member 9; GSTP1 means Glutathione S-Transferase Pi 1; P less than 0.05 means statistical significance.

### Influence of donor VIPDEGs gene polymorphisms on tacrolimus pharmacokinetics

To understand the mechanism underlying the SFTC. The affections of VIPDEGs gene polymorphisms on tacrolimus metabolism were further investigated. The influence of twelve SNPs genotypes of four VIPDEGS on tacrolimus logCDR at different times after LT was individually analyzed using PLINK (**Table 4**). Our results revealed three SNPs that showed significant association with SFTC were predictors of tacrolimus logCDR at every week (all *p* < 0.05).

**Table 4:** The association between SNPs of VIPDEGs and tacrolimus pharmacokinetics at the first month after LT.

p-value of each week								
SNP	Gene	Genotype (Frequency,n)			1st	2nd	3rd	4th
rs28365085	*CYP3A5*	GG	GA	AA	0.02	0.41	0.11	0.07
		0	4	110				
rs55965422	*CYP3A5*	GG	GA	AA	0.42	0.86	0.74	0.69
		0	2	112				
rs4646450	*CYP3A5*	AA	AG	GG	<0.001	0.03	0.02	<0.001
		8	52	53				
rs6977165	*CYP3A5*	CC	CT	TT	0.00	0.02	0.01	0.049
		0	6	108				
rs776746	*CYP3A5*	AA	AG	GG	<0.001	0.05	0.02	<0.001
		8	51	54				
rs1800073	*CFTR*	TT	TC	CC	0.13	0.08	0.04	0.53
		0	2	112				
rs141723617	*CFTR*	CC	CT	TT	0.17	0.71	0.86	0.46
		0	3	111				
rs121909046	*CFTR*	GG	GA	AA	0.09	0.80	0.96	0.63
		0	4	110				
rs213950	*CFTR*	AA	AG	GG	0.05	0.19	0.01	0.09
		23	44	47				
rs75789129	*CFTR*	GG	GA	AA	0.75	0.69	0.47	0.26
		0	12	102				
rs1057910	*CYP2C9*	CC	CA	AA	0.77	0.78	0.03	0.10
		0	8	106				
rs1695	*GSTP1*	GG	GA	AA	0.72	0.34	0.76	0.87
		6	34	74				

CYP3A5 means cytochrome P450 3A5; CFTR means CF Transmembrane Conductance Regulator; CYP2C9 means Cytochrome P450 family 2 subfamily C member 9; GSTP1 means Glutathione S-Transferase Pi 1; P less than 0.05 means statistical significance.

Besides, different genotype groups based on whether carrying the SFTC-related allele were compared. In *CYP3A5* rs4646450 carriers, the tacrolimus logCDR was lower in the A/A or A/G group than that of the G/G group each week (*p* < 0.05) (**Figure 2C**). As for *CYP3A5* rs6977165, the C/T group was showed lower tacrolimus logCDR than T/T group at each week (*p* < 0.05) (**Figure 2D**). And in the A/A or A/G group of *CYP3A5* rs776746, tacrolimus logCDR was lower than that of the G/G group at weeks 1, 3 and 4, respectively (*p* < 0.05) (**Figure 2E**).

## Discussion

In this study, we found that SFTC increased the incidence of IR and dyslipidemia. Besides, rs4646450, rs6977165, and rs776746 in *CYP3A5* were associated with SFTC, the reason might be that rs4646450, rs6977165, and rs776746 were correlated with tacrolimus pharmacokinetics.

Most liver transplant recipients receive either tacrolimus or cyclosporine to prevent rejection, while tacrolimus is the first line in immunosuppressive regimens (11). Tacrolimus has a better immunosuppressive effect and fewer side effects as compared to cyclosporine **(** 12–14 **)**. A TMC randomized controlled trial suggested that tacrolimus-based immunosuppression is preferable to cyclosporine during the first year after LT (15). And the conversion from cyclosporine to tacrolimus has been reported to improve the cardiovascular risk profile and may retard further decline of renal function after LT (16). However, some LT recipients had to change the immunosuppressive agents from tacrolimus to cyclosporine because of rapid tacrolimus metabolism or diabetes **(** 17 **–** 18 **)**. Previous studies have shown that this kind of conversion can release adverse reactions in patients to some extent with no increased risk of rejection **(** 19 **–** 20 **)**, which is also found in our study, but we found SFTC increased the incidence of IR and dyslipidemia. Importantly, patients with SFTC also indicate a low dose of tacrolimus at the initial dose, which is prone to acute rejection. Hence it makes sense to know whether LT recipients were suitable for using FK506 at the beginning.

In our study, patients with unstable tacrolimus blood concentration and hyperglycemia were converted to cyclosporine, and these patients were thought to be intolerant to tacrolimus. To better understand the mechanism of tacrolimus intolerance, transcriptome data analysis was performed to identify an expression profile of the very important pharmacogenetic genes between the SFTC group and NSFTC group. Four VIP genes (*CYP3A5*, *CFTR*, *CYP2C9*, *GSTP1*) were found to be up-regulated in the SFTC. The influence of gene polymorphisms of these four genes on the SFTC and tacrolimus metabolism were then investigated. Three SNPs (rs4646450, rs6977165, rs776746) of *CYP3A5* were found to be the potential risk factors of SFTC. To further investigate the potential mechanism, we further explored the relationship between these SNPs and tacrolimus metabolism at different time points after LT. The results showed that the three SNPs related to SFTC had a statistically significant association with the tacrolimus logCDR every week after LT. When grouping by different genotypes, it was found that individuals carrying the SFTC-related allele had lower logCDR values than those not carrying it. These results suggest that rs4646450, rs6977165, and rs776746 were the independent risk factor of tacrolimus intolerance through the influence of tacrolimus metabolism. To the best of our knowledge, this is the first time to explore the potential factors of tacrolimus intolerance after LT.

Cardiovascular disease, a contributor to the elevated mortality rate among liver transplant recipients, is an adverse consequence of metabolic syndrome (visceral obesity, dyslipidemia, hyperglycemia, and hypertension). **(** 21 **,** 22 **),** In our study, SFTC increases the incidence of dyslipidemia after LT, which is consistent with the previous studies that tacrolimus appears less likely to cause hypercholesterolemia than cyclosporine **(** 23 **,** 24 **)**. Although a direct correlation between SFTC and new-onset diabetes was not observed, SFTC was associated with insulin resistance appearing in the fourth week after LT, the latter was a potential risk factor for new-onset diabetes.

Enzymes in the cytochrome P450 (CYP) 3A family are responsible for the oxidative metabolism of tacrolimus or other drugs in various human organs and tissues, especially in the liver **(** 25 **,** 26 **)** *CYP3A5*, encoding the cytochrome P450 (CYP) enzymes 3A5, is the most influential gene in tacrolimus pharmacokinetics by far (27) and *CYP3A5* rs776746 polymorphism is the most well-known found to be related to tacrolimus pharmacokinetics. Patients can be divided into *CYP3A5* expressers carrying at least one CYP3A5*1 allele and *CYP3A5* non-expressers with CYP3A5*3/*3 genotype based on the *CYP3A5* rs776746 genotype (28). And the former requires a higher maintenance tacrolimus dose to achieve the target tacrolimus concentration as compared to the latter (29), which is also found in our study. As for *CYP3A5* rs4646450, there have been several studies that pointed out that it might be associated with tacrolimus metabolism **(** 30 **,** 31 **)**. However, there are no previous reports on the effects of *CYP3A5* rs6977165 polymorphism on tacrolimus metabolism. In our study, we found that donor rs4646450, donor rs6977165, and donor rs776746 polymorphism were the independent risk factors with an increased risk of >3-fold of tacrolimus intolerance by each risk allele. And all three SNPs showed a significant association with tacrolimus pharmacokinetics in the first four weeks.

Several limitations existed in our study. Firstly, the limitation of the donor demographic data may influence the result because the expression data stemmed from the donor liver samples. But the quality of donors’ livers strictly conformed to the standards of transplantation which in a way offset some of our concerns. Secondly, the result may be controversial because of the small sample size, further study will be needed by using a larger data set. Lastly, we only focus on the donor genotypes in our study since no recipient expression data is available. It is also necessary to apply this workflow to recipient samples in future studies.

In conclusion, this study demonstrated that SFTC is a potential risk factor for dyslipidemia and IR. Besides, donor *CYP3A5* rs4646450, *CYP3A5* rs6977165, and *CYP3A5* rs776746 genotype might relate to tacrolimus intolerance in Chinese liver transplant recipients by affecting tacrolimus metabolism, among which donor rs6977165 is the first time suggested correlated with tacrolimus metabolism. This information may be useful to obtain personalized medicine earlier, avoid the incidence of adverse effects and relieve medical care burdens for LT patients.

## Data availability statement

The datasets presented in this study can be found in online repositories. The names of the repository/repositories and accession number(s) can be found below: https://www.ncbi.nlm.nih.gov/, GSE53792.

## Ethics statement

The study was approved by the Ethics Committee of Shanghai First Hospital affiliated with Shanghai Jiao Tong University. These methods are based on the Helsinki Declaration and its subsequent amendments or similar ethical standards. The patients/participants provided their written informed consent to participate in this study.

## Author contributions

Conceptualization: ZP, JF, and HP. Methodology: YL, RW, and PW. Data collection: JZ, TZ, PZ, and FZ. Visualization: RW, PW, and YL. Funding acquisition: ZP. Writing – original draft: YL, RW, and PW. Writing – review & editing: YL , WA and HW. All authors contributed to the article and approved the submitted version.

## Funding

This work was supported by the National Natural Science Foundation of China (82070677).

## Conflict of interest

The authors declare that the research was conducted in the absence of any commercial or financial relationships that could be construed as a potential conflict of interest.

## Publisher’s note

All claims expressed in this article are solely those of the authors and do not necessarily represent those of their affiliated organizations, or those of the publisher, the editors and the reviewers. Any product that may be evaluated in this article, or claim that may be made by its manufacturer, is not guaranteed or endorsed by the publisher.

## Supplementary material

Supplementary Figure 1 The influence of SFTC to survival. Kaplan-Meier survival curves analysis of patients between the SFTC and NSFTC groups; SFTC means switching from tacrolimus to cyclosporine; P less than 0.05 means statistical significance.

Supplementary Figure 2 The correlation of complications and survival. **(A)** Kaplan-Meier survival curves analysis of patients between the rejection and no-rejection groups; **(B)** Kaplan-Meier survival curves analysis of patients between hypertension and no-hypertension groups; **(C)** Kaplan-Meier survival curves analysis of patients between IR and no-IR groups; **(D)** Kaplan-Meier survival curves analysis of patients between dyslipidemia and no-dyslipidemia groups. IR means insulin resistance; P less than 0.05 means statistical significance.

## Abbreviations

LT, liver transplantation; SFTC, switching from FK506 to cyclosporine; NSFTC, non-switching from FK506 to cyclosporine; SNP, single nucleotide polymorphism; DCD, donations after cardiac death; CDR, trough blood concentration/weight-adjusted-dose ratios; EXON, Infinium Human Exome-12 v1.2 BeadChip; VIP, the very important pharmacogenetic genes; PSM, propensity score matching; logCDR, CDR normalized by logarithmic transformation; VIPDEGs, differentially expressed VIP genes; MAF, minor allele frequency; HWE, Hardy–Weinberg Equilibrium; OR, odds ratio.
