# Supplementary figures and images for: Genetic factors underlying tacrolimus intolerance after liver transplantation
Source: Front Immunol. 2022 Sep 30;13:944442. doi: 10.3389/fimmu.2022.944442 (PMC9562471; doi:10.3389/fimmu.2022.944442)

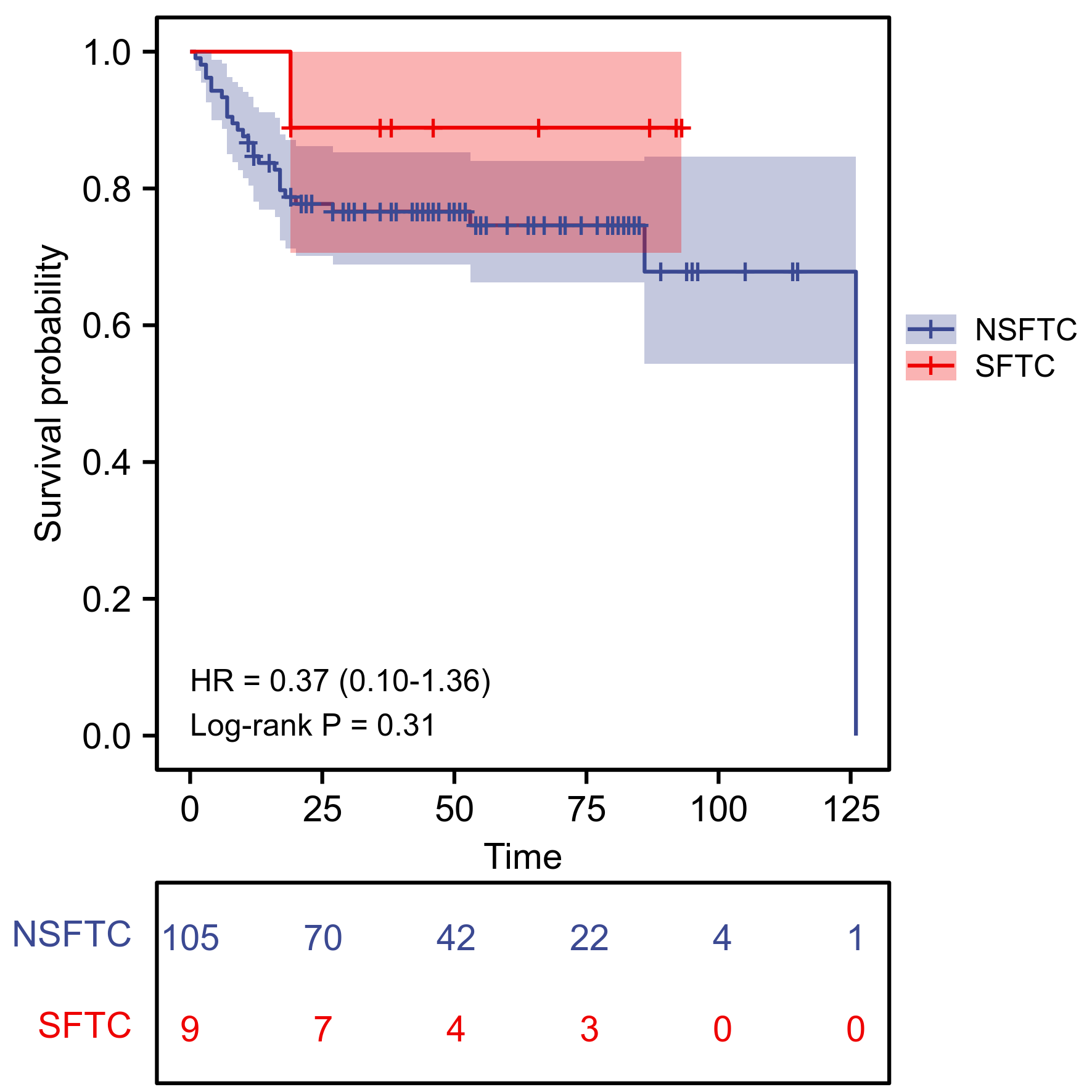

Supplement: Supplementary Figure 1 — The influence of SFTC to survival. Kaplan-Meier survival curves analysis of patients between the SFTC and NSFTC groups; SFTC means switching from tacrolimus to cyclosporine; P less than 0.05 means statistical significance. [file Image_1.tiff]

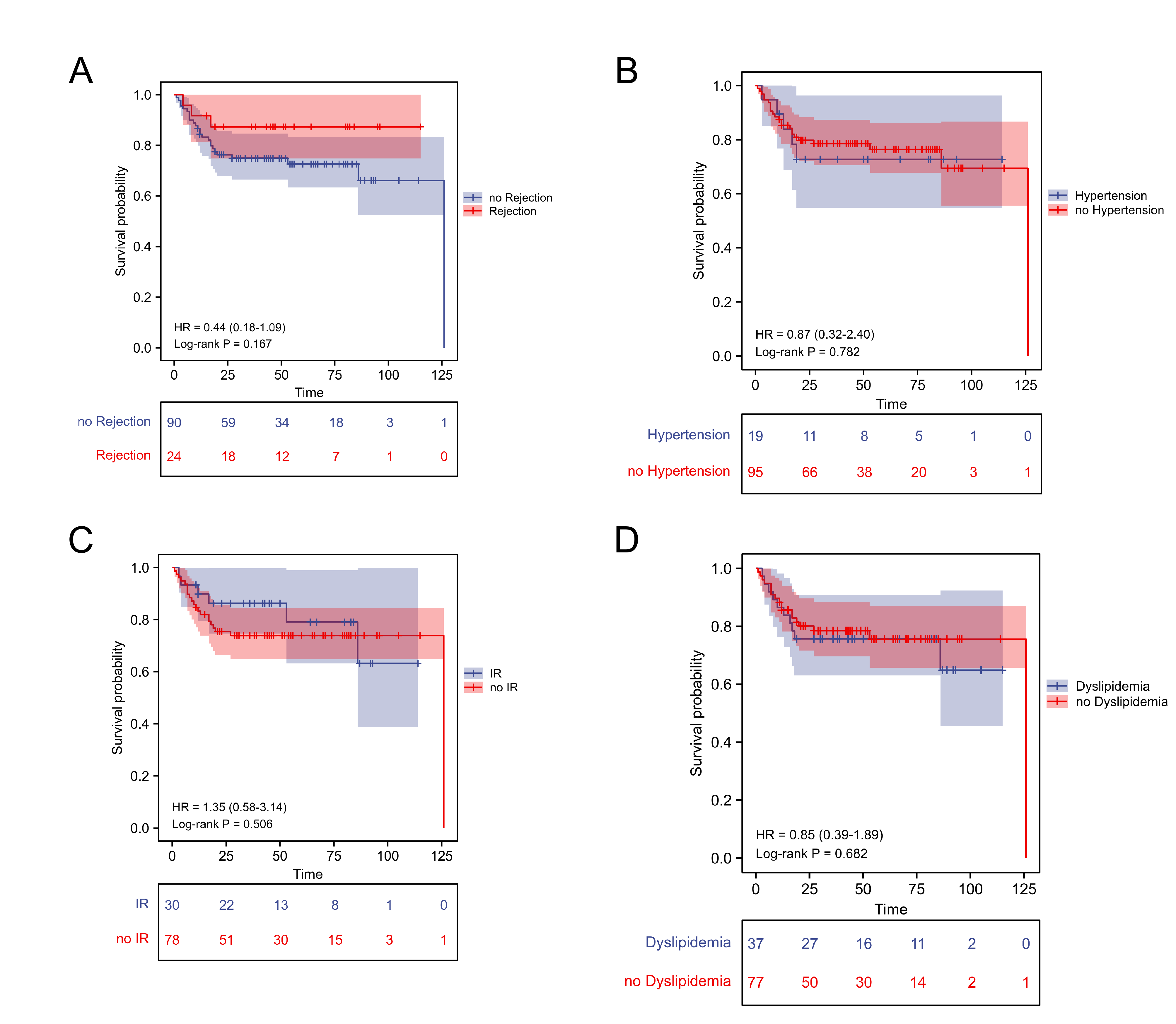

Supplement: Supplementary Figure 2 — The correlation of complications and survival. (A) Kaplan-Meier survival curves analysis of patients between the rejection and no-rejection groups; (B) Kaplan-Meier survival curves analysis of patients between hypertension and no-hypertension groups; (C) Kaplan-Meier survival curves analysis of patients between IR and no-IR groups; (D) Kaplan-Meier survival curves analysis of patients between dyslipidemia and no-dyslipidemia groups. IR means insulin resistance; P less than 0.05 means statistical significance. [file Image_2.tiff]
